# Hemoglobinopathies in the North of Morocco: Consanguinity Pilot Study

**DOI:** 10.1155/2019/6857417

**Published:** 2019-09-26

**Authors:** Achraf Laghmich, Fatima Zahra Alaoui Ismaili, Zeineb Zian, Amina Barakat, Naima Ghailani Nourouti, Mohcine Bennani Mechita

**Affiliations:** Biomedical Genomics and Oncogenetics Research Laboratory, Faculty of Sciences and Techniques of Tangier, University Abdelmalek Essaâdi, Tangier 90000, Morocco

## Abstract

Consanguinity is a social behavior characterized by the arrangement of marriages between relatives. It coincides generally with the geographic distribution of recessive genetic diseases as it increases the likelihood of homozygosis and, consequently, the incidence of their pathologies in the population. In this pilot study, we assess the effect of inbreeding on the burden of hemoglobinopathies in Northern Morocco. From January 2016 to December 2018, 197 children born in the studied region to three ancestral generations and diagnosed with hemoglobinopathies were subject to investigation. The rate of consanguinity in the parents' generation of children with hemoglobinopathies was 50.25%, with first cousin marriages accounting for 68.69% of consanguineous unions (FI = 0.02). The corresponding rates in the general population, based on a sample of *N* = 900, were 29.67% and 82.02%, respectively. The marriages between first cousins are the most common among the other types of consanguineous unions. Our study propounds that consanguinity substantially contributes to the hemoglobinopathy burden in the studied region and has changed little over time. Refraining from consanguineous marriages and detecting couples at risk could contribute to the reduction of the incidence of genetic diseases in our country.

## 1. Introduction

Consanguineous marriages have been practiced since the early existence of mankind. Many assume that their rate declines with modernization and increased literacy; yet this is not supported by scientific statistics [1, 2]. At present, consanguinity is widely popular in many parts of the world. Eleven percent of the global population is married to a kinsperson [3]. The Middle East and North Africa are known for high levels of consanguineous kinships compared to Western countries [4]. In Morocco, consanguinity varies in the range of 19.81–28% [5]. Regional studies showed that the northern regions register higher inbreeding rates. Recent studies have evaluated consanguinity in the global population of the Tangier-Tetouan region as 39.4% and of the Tangier-Tetouan-Al Hoceima region as 24.37% [5, 6]. Consanguinity in this region seems to be characterized by specific features of socioeconomic, cultural, historical, and geographical order [7]. Given our insufficient knowledge on the impact of consanguinity on genetic diseases in Morocco, our study aims to assess the rate of inbreeding in hemoglobinopathy population of Northern Morocco. Recent researches have highlighted a high prevalence of this group of inherited hemoglobin disorders in the studied region. Their severity and disabling nature make them a major public health problem [8].

## 2. Population and Methods

During January 2016 and December 2018, inbreeding was studied in the paediatrics services in 197 children already diagnosed with one or more defects responsible for hemoglobinopathies. The participants from the studied region were of at least three ancestral generations. The study population consisted of 100 females and 97 males aged between 0 and 15 years. All the parents were well informed and gave consent. Individual interviews were done using preestablished questionnaires inquiring about nationality, sex, age, dialect, nativity, and data relative to the consanguinity of the parents and grandparents. Questionnaires and written consent forms were available in Arabic to ensure a comprehensive understanding of the study objectives.

**Figure 1:**
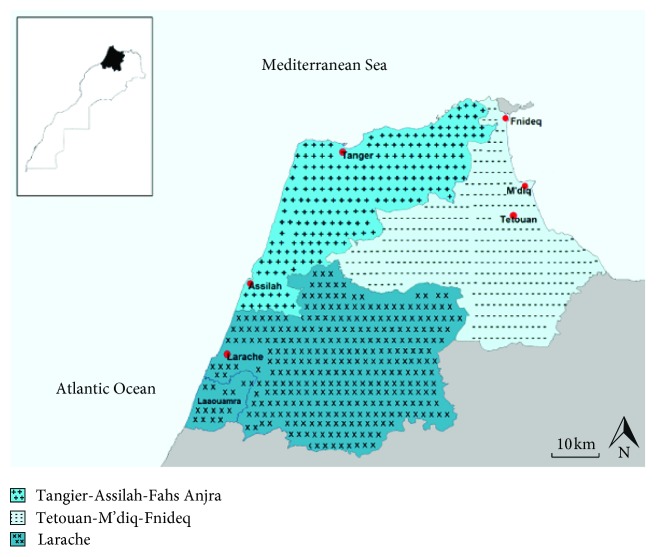
Geographic location of the studied region.

The studied region is located in the north of Morocco along the southern Mediterranean coast of the Thalassemia Belt. It covers an area of 7098.8 km^2^ and has a population of 2,386,852 inhabitants [9].  Figure 1 shows the geographic location of the studied region divided into three centers: Tangier-Assilah Province, Tetouan-M'diq-Fnideq Province, and Larache Province.

The present study investigates the types and rate of consanguinity, its coefficient (*F*), and its relationship with hemoglobinopathies in the studied region. Consanguineous marriages were classified according to the *F* which is the probability that two equivalent genes are identical by descent [10]. The mean coefficient of inbreeding (FI) in the population is the sum of the *F* of its individuals:

(1) $$\begin{array}{c} \text{FI}=\sum {\left(\frac{1}{2}\right)}^{\left(m+p+1\right)}, \end{array}$$

where *m* = number of generations connecting the mother of an individual to the common ancestor and *p* = number of generations connecting the father of an individual to the common ancestor.

**Figure 2:**
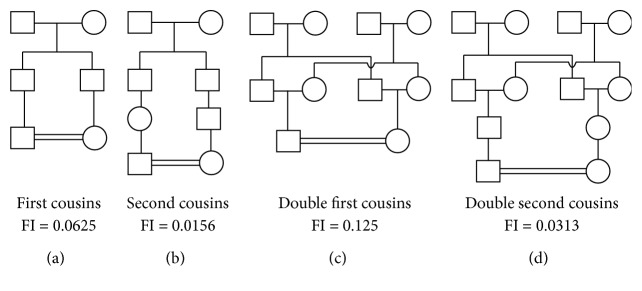
Consanguineous marriages: types and coefficient of inbreeding of descendants.

The biological relationships were classified into one of the following categories: first cousin, double first cousin, second cousin, double second cousin, and nonconsanguineous (Figure 2).

An independent-sample test was carried out on 900 healthy children aged between 0 and 15 years to assess the consanguinity rate in the global population of the studied region. The consanguinity/disease relationship was studied using the chi-square test. The odds ratio (OR) test was used to confirm the result by the following formula:

(2) $$\begin{array}{c} \text{OR}=\frac{\left(a/c\right)}{\left(b/d\right)}, \end{array}$$

where *a* is exposed cases that are consanguineous children in the hemoglobinopathy population; *b* is exposed controls that are consanguineous children in the general population; *c* is not exposed cases that are not consanguineous children in the hemoglobinopathy population; and *d* is not exposed controls that are not consanguineous children in the general population.

Our data were analyzed using SPSS statistical software version 11.5. Statistical significance was measured at *p* < 0.05.

## 3. Results

The population of the studied region is mainly Muslim. 100% of our sample were declared to be Muslim and Arabic speakers. All participants perceived the advantage of consanguineous marriages by eliminating social risk and offering security to women and children by strengthening family ties. Our results revealed a highly significant difference in the rates of consanguineous marriages between the global population and children with hemoglobinopathies. A mild difference was observed between the current generation, the “parents' generation,” and the previous generation of grandparents. No significant difference between the paternal and maternal grandparents was observed. The mean rate is given in Tables 1 and 2. Consanguinity was estimated in hemoglobinopathy population as 50.25% in parents and as 64.47% in grandparents. In the global population, the rate of consanguinity went through a mild increase. It amounted to 22.55% and 29.67% in grandparents and parents' generations, respectively. The same distribution was found between the three provinces in the consanguinity of the hemoglobin disorders and the global population (Tables 1 and 2).

First cousins marriages were the most common among parents with children suffering from hemoglobinopathies. They represented more than half of the consanguineous marriages in both generations, followed by marriages between second cousins. The frequency of other marriages was very low (Table 3). 131 (66.5%) families resided in rural areas and 66 (33.5%) in urban areas. The inbreeding was estimated in the urban environment as 46.97% (FI = 0.0312) and in the rural areas as 51.91% (FI = 0.0297) (Table 4).

The consanguinity rate was significantly higher in people affected by sickle cell anemia (52.08%) than thalassemia (45.28%). Hemoglobinopathies were found depending on consanguinity in the studied region (*p* < 0.05). The same result was confirmed by the odds ratio test (OR = 2.31 > 1). Larache Province registered the weakest dependence rate (Table 5).

## 4. Discussion

Consanguineous marriages are culturally favored in Morocco, and they are an integral part of the social life of the country. To date, only scarce works have studied consanguinity in Morocco. They were mainly limited to some regions of Morocco or reflected only the activity of a specific medical center [11, 12]. No study on consanguinity in hemoglobinopathy population has been reported despite its importance. Our research was the first to assess the rate of consanguinity in hemoglobinopathy population in our country. This rate was evaluated as 50.25% (FI = 0.02). It was significantly higher than the rate of consanguinity in the general population in the same area evaluated as 29.66% (FI = 0.01) (Table 6). The high rate of this practice in couples with offspring affected by autosomal recessive conditions was already reported in many studies. Consanguinity is believed to increase the likelihood of homozygosis by common descent and thereby the incidence of recessive genetic diseases in the population [11]. It can increase by eightfold the burden of some hemoglobinopathies in the population [12]. The probability of practicing this type of marriage in the region is far from random and depends on several economic, sociocultural, and demographic factors. Studies have shown that consanguineous marriages are favored to eliminate social risk and to provide security to women and children by strengthening family ties and preserving wealth [13, 14].

Previous works showed that the national consanguinity rate in Morocco varied between 19.81 and 28% [6]. Northern regions registered the highest rates with 39.4% (FI = 0.02033) in the Tangier-Tetouan region [6] and 24.37% (FI = 0.008038) in the Tangier-Tetouan-Al Hoceima region [4]. The Gharb-Chrarda-Beni Hssen region, the neighbor region, also registered a high rate of consanguinity (22.8%) [15].

Compared to other Arab countries, Morocco registers the lowest rates of consanguinity. This rate is estimated as 34% in Algeria, 39.3% in Tunisia, 47.2% in Mauritania, 48.4% in Libya, 54.2% in the UAE, 54% in Qatar, and 66.7% in Saudi Arabia [5].

Our results showed that consanguinity is increasing in the global population (with 22.55% in the previous generation against 29.66% in the current one). Scientific literature has been describing big changes in Morocco regarding consanguinity throughout time. In 1994, Bouazzaoui et al. indicated a decreasing consanguinity rate, while more recent studies showed an increasing rate (21.5% to 25.4% in the present generation) [7, 11]. The survival of this tradition in the studied region is very likely in the next generations. Consanguinity rates seem to continue being high in hemoglobinopathy population among the general population because it is generally accepted that its social advantages outweigh the disadvantages. This tradition still finds its origin in motivations of socioeconomic and cultural order. The participants perceived the advantage of this type of marriage in promoting family stability and maintaining family properties. This was already discussed in other Arab populations (Qatar, Yemen, UAE, and Algeria) [16–18].

On the contrary, a decrease in the rate of consanguineous marriages was observed in some other populations (Jordan, Lebanon, Bahrain, and Palestine) as a result of the increasing female education, the declining fertility resulting in less relatives, more urbanization of populations, and the improving economic status of families [13, 16].

In hemoglobinopathy population, results revealed a significant decrease (14.22%) in the overall prevalence of consanguineous marriages between the previous and the current generation (64.47% vs 50.25%). Significant decreases were already reported in other populations with hemoglobinopathies as a result of premarriage counseling and awareness of the general population about hemoglobinopathies [19, 20]. Numerous focused awareness campaigns could be behind this decrease in our studied region. First cousin marriages were found to be the most common; they represent almost two-thirds of the consanguineous marriages in both generations. Marriages between second cousins were estimated as 25.25% in parents' generation and 22.83% among grandparents. The frequency of other marriages was low (Table 3). These results were in agreement with others findings reporting a high rate of consanguinity in hemoglobinopathy population due to first cousin marriages that constitute more than 50% of the consanguineous marriages [12, 21–23].

Inbreeding in urban areas seems to differ from that in rural areas. It was estimated as 46.97% (FI = 0.0312) in urban areas and 51.91% (FI = 0.0297) in rural ones. Previous studies showed that consanguinity rates were lower in urban areas when compared to those in rural ones where the ownership of agricultural land and the work of the land are decisive factors. Living in difficult socioeconomic conditions significantly increases the probability of occurring consanguineous marriages [24, 25].

In our hemoglobinopathy population, first cousin rates in urban and rural areas were 37.88% and 32.82%, respectively. This rate was already reported in general populations to be lower in urban than in rural areas. It was evaluated in Algeria as 10% and 15% [25], in Egypt as 8.3% and 17.2% [26], and in Jordan as 29.8% and 37.9% [27], respectively. The high rate of consanguinity in our rural areas was due to the abundance of second cousin unions (16.03% to 6.06% in urban areas). This could be a result of the genetic literacy in this area where second cousin unions were seen as nonconsanguineous. More efforts have to be made in this area.

The inbreeding rate was significantly higher in sickle cell anemia (52.08%) than thalassemia (45.28%). This could be explained by the concentration of sickle cell anemia patients in rural areas in the three provinces. Consanguinity rates were reported higher in these settings because of many socioeconomic factors [5, 15, 28].

Hemoglobinopathies were found to be dependent on consanguinity in our studied region (*p* < 0.05). This dependence was strong in Tangier-Assilah-Fahs Anjra Province and in Tetouan-M'diq-Fnideq Province but weaker in Larache Province. This could be explained by the high incidence of thalassemia in this province [8], and the abundance of thalassemic children born to nonconsanguineous marriages (Table 5). In fact, the risk of having a thalassemic offspring is estimated to be high even in distant relationship marriages because of the high gene frequency in the population.

Consanguinity contributes considerably to aggravate the burden of hemoglobinopathies in the studied region. Avoiding this habit is not enough to eradicate hemoglobinopathies in this area. The implementation of an obligatory premarital screening program for all couples at risk could also have a significant impact on decreasing this incidence [29, 30]. A recent study performed at a marriage center in Saudi Arabia showed that more than 60% of the participants at high-risk marriages annulled their marriage proposals. This reduced the incidence of sickle cell anemia and other genetic diseases across the region [12, 15]. Cultural pressure and traditions could be an obstacle for the eradication of consanguinity in Morocco. This has been already highlighted in some of the population where no significant decrease in consanguinity was observed, since almost 90% of the high-risk couples married each other. The introduction of genetic screenings during secondary school, before wedding plans are formed, is recommended as these plans are difficult to be canceled because of cultural concerns [22, 31].

## 5. Conclusion

The geographic distribution of hemoglobinopathies overlaps with consanguinity in our studied region. Public awareness of genetic risks related to consanguineous marriages and premarital genetic counseling are necessary. Our results have enriched the literature of consanguinity among the Northern Moroccan population and its relation to hemoglobinopathies in this region. Because of the high cost of treatment, difficulties in the follow-up, and the low income of the population, avoiding consanguineous unions and detection of couples at risk could be an elementary prevention strategy to reduce the incidence of hemoglobinopathies in Morocco.

## Figures and Tables

**Table 1:** Distribution of inbreeding types in the general population and hemoglobinopathy population in parents.

Type of consanguinity	General population		Hemoglobinopathy population	
	*N*	%	*N*	%
Double first cousin	10	1.11	3	1.52
First cousin	219	24.33	68	34.52
Double second cousin	8	0.89	3	1.52
Second cousin	30	3.33	25	12.69
Consanguineous	267	29.67	99	50.25
Nonconsanguineous	633	70.33	98	49.75
Total	900	100.00	197	100.00
FI	**0.01**		**0.02**	

*N* = number; % = frequency; FI = coefficient of consanguinity.

**Table 2:** Distribution of consanguinity in hemoglobinopathy population in the studied region.

Province	*N*	Parents				Grandparents			
		C		NC		C		NC	
		*N*	%	*N*	%	*N*	%	*N*	%
Tangier-Assilah	35	19	9.64	13	6.60	20	10.15	12	6.09
Tetouan-M'diq-Fnideq	32	21	10.66	14	7.11	25	12.69	10	5.08
Larache	130	59	29.95	71	36.04	82	41.62	48	24.37
Total	197	99	50.25	98	49.75	127	64.47	70	35.53

C = consanguineous; NC = nonconsanguineous; *N* = number; % = frequency.

**Table 3:** Types of inbreeding in hemoglobinopathy population in the studied region.

Province	Parents					Grandparents		
	DFC	FC	DSC	SC	DFC	FC	DSC	SC
Tangier-Assilah	0	14	0	5	0	16	0	4
Tetouan-M'diq-Fnideq	1	15	1	4	1	18	0	6
Larache	2	40	2	15	3	60	0	19
Studied region, *N* (%)	3 (3.03)	68 (68.68)	3 (3.03)	25 (25.25)	4 (3.14)	94 (74.01)	0 (0)	29 (22.83)

DFC = double first cousin; FC = first cousin; DSC = double second cousin; SC = second cousin; *N* = number; % = frequency.

**Table 4:** Distribution of inbreeding types in hemoglobinopathy population by place of residence.

	Studied region		Urban		Rural	
	*N*	%	*N*	%	*N*	%
Total number	197	100.00	66	100.00	131	100.00
No relationship	98	49.75	35	53.03	63	48.09
Consanguineous	99	50.25	31	46.97	68	51.91
Double first cousin	3	1.52	1	1.52	2	1.53
First cousin	68	34.52	25	37.88	43	32.82
Second cousin	25	12.69	4	6.06	21	16.03
Double second cousin	3	1.52	1	1.52	2	1.53

*N* = number; % = frequency.

**Table 5:** Independence of inbreeding and hemoglobinopathies in the studied area.

	Global population		Hemoglobinopathies		*χ* ^2^	*p* value (%)
	C	NC	C	NC		
Tangier-Assilah	77	223	19	13	15.98	0
Tetouan-M'diq-Fnideq	86	214	21	14	14.15	0
Larache	104	196	59	71	4.42	4
Studied region	267	633	99	98	30.81	0

*C* = consanguineous; NC = nonconsanguineous.

**Table 6:** Comparison of the consanguinity profile of Moroccan population.

Type of study	Population study	Reference, Year (*N*)	Consanguinity rate (%)	FI	FC + DFC (%)	DSC + SC (%)
Regional	Families with hemoglobinopathies in Northern Morocco	This study, 2018 (197)	50.25	0.02	36.04	14.21
Regional	Northern Morocco	This study, 2018 (900)	29.66	0.01	25.44	4.22
Regional	Tangier-Tetouan region	Hardouz et al., 2014 (160) [6]	39.4	0.02	30	5.6
National	Families with autosomal recessive diseases in Rabat	Cherkaoui Jaouad et al., 2009 (176) [12]	59.09	0.03	43.18	11.93
National	All Moroccan regions	Bouazzaoui, 1994 (4773) [11]	19.90	0.008	No data	No data
Regional	Different regions	Talbi, 2007 (873) [7]	22.79	0.008	9.69	4.93^*∗*^

FI = coefficient of inbreeding; DFC = double first cousin; FC = first cousin; DSC = double second cousin; SC = second cousin. ^*∗*^Double second cousins are not included in this prevalence.

## Data Availability

The data used to support the findings of this study are available from the corresponding author upon request.
